# Pomegranate and Its Components, Punicalagin and Ellagic Acid, Promote Antidepressant, Antioxidant, and Free Radical-Scavenging Activity in Ovariectomized Rats

**DOI:** 10.3389/fnbeh.2022.836681

**Published:** 2022-05-06

**Authors:** Nancy Cervantes-Anaya, Gabriel Azpilcueta-Morales, Erika Estrada-Camarena, Daniela Ramírez Ortega, Veronica Pérez de la Cruz, Maria Eva González-Trujano, C. López-Rubalcava

**Affiliations:** ^1^Laboratorio de Neuropsicofarmacología, Dirección de Neurociencias, Instituto Nacional de Psiquiatría Ramón de la Fuente Muñiz, Secretaria de Salud (SSA), Mexico City, Mexico; ^2^Laboratorio de Neurobioquímica y Conducta, Instituto Nacional de Neurología y Neurocirugía Manuel Velasco Suárez, Secretaria de Salud (SSA), Mexico City, Mexico; ^3^Laboratorio de Neurofarmacología de Productos Naturales, Dirección de Neurociencias, Instituto Nacional de Psiquiatría Ramón de la Fuente Muñiz, Secretaria de Salud (SSA), Mexico City, Mexico; ^4^Laboratorio 16, Departamento de Farmacobiología, Centro de Investigación y Estudios Avanzados, Mexico City, Mexico

**Keywords:** antioxidant, antidepressant-like effect, aqueous extract of *Punica granatum* (AEPG), punicalagin, ellagic acid, ovariectomy, free radical-scavenging activity, pomegranate

## Abstract

Previous reports described the antidepressant-like action of the aqueous extract of pomegranate (*Punica granatum*: AEPG). Thus we evaluated the effect of AEPG and the main compounds found in the extract, punicalagin (PNCG) and ellagic acid (EA), on forced swimming test and the redox environment (reactive oxygen species [ROS] production, lipoperoxidation [LPX], and cellular function) in the brain of rats treated with 3 weeks post ovariectomy exposed *ex vivo* to pro-oxidants. Also, we selected PNCG and EA to study their antidepressant-like effects (0.001, 0.01, 0.1, 1.0, and 10 mg/kg) in the forced swimming test and their scavenging capacities in chemical combinatorial assays (expressed as IC_50_ values). We observed a 2-fold increase in the formation of ROS and LPX in the brain after exposure to FeSO_4_. However, these effects were significantly attenuated when rats were treated with AEPG, PNCG, and EA (1 mg/kg and 0.010 mg/kg for 14 days). AEPG and EA significantly increased the cellular function values of brains that had been affected by the effect of FeSO_4_ and with ONOO^–^. PNCG and EA significantly reduced immobility behavior at the lower doses used in this study. The capacity of scavenging compounds to eliminate radicals was for hydroxyl radical (^⋅^OH), superoxide anion (O2^⋅⁣–^), and peroxynitrite (ONOO^–^) as follows: AEPG > punicalagin > ellagic acid. In conclusion, the AEPG and their active compounds PNCG and EA promote antidepressant-like actions and antioxidant activity as they attenuate oxidative damage and prevent cellular dysfunction in ovariectomized rat brains.

## Introduction

Depression is a psychiatric disorder that involves genetic and epigenetic factors that affect up to 20% of the population worldwide (Nestler et al., 2002; Scapagnini et al., 2012). Depression is one of the most important public health problems that impact over 25% of women and 12% of men (Yan et al., 2010), being of greater risk for women (Birmaher et al., 1996; Soares, 2013), particularly within their reproductive life (Bautista et al., 2012). Indeed, vulnerability to depression increases during the transition to menopause, and reproductive aging is associated with an increased risk of depression (Maki et al., 2010, 2012; Bautista et al., 2012; Soares, 2013).

Changes in the redox balance are involved in the pathogenesis of depressive disorders. The brain is vulnerable to oxidative stress because it has a high metabolic rate and, consequently, a high oxygen consumption (20% of all the body oxygen) (Topçuoglu et al., 2009; Di Lorenzo et al., 2016). The brain is also susceptible to lipid peroxidation (LPX) as it has polyunsaturated fatty acids as the main component of neuronal cell membranes, which are susceptible to free radicals, and they present high levels of iron, which is a highly pro-oxidant compound (Valko et al., 2007). Patients with depression present a relatively poor antioxidant defense (Bhatt et al., 2020) and have significant low plasma levels of antioxidants, such as vitamin E, coenzyme Q10, tryptophan, tyrosine, estrogens, zinc, and albumin together with an overall reduction in total antioxidant capacity (Maes et al., 2011; Bhatt et al., 2020). Increased reactive oxygen species (ROS) is known to cause lipid peroxidation in the brain cell membrane, resulting in the production of the oxidative stress marker compound malondialdehyde (MDA), and clinical studies reported that serum MDA levels in patients with depression were higher than in a control group (Birmaher et al., 1996; Sarandol et al., 2007). For these reasons, the redox balance is currently considered an important event in depression etiology.

It has been reported that in oxidative stress is increased in postmenopausal women (Sánchez-Rodríguez et al., 2017, 2019). Similar observations are derived from animal models, where a menopause-like condition is induced by ovariectomy (Finch, 2014). Reports indicate that ovariectomy increases lipid peroxidation in the brain and erythrocytes, increases proinflammatory cytokine production, and decreases the antioxidant activity of glutathione peroxidase (Yazğan et al., 2016). Also, OVX produces neuron cell damage by decreasing cell viability in the hippocampus, as well as increasing malondialdehyde (MDA) levels and decreasing superoxide dismutase (SOD) activity (Zhou et al., 2021). Interestingly, estradiol and the phytoestrogen Biochanin A prevents these effects (Yazğan et al., 2016; Zhou et al., 2021). Together, these data suggest that a menopause-like condition induced by ovariectomy is a factor that favors a pro-oxidant status.

Currently, 30% of depressed patients do not respond to existing pharmacological therapies, and the remaining 70% do not achieve complete remission (Scapagnini et al., 2012; Pathak et al., 2013). Hence, finding new drugs for the treatment of depression is highly necessary (Girish et al., 2012). Furthermore, some of the new drugs with antidepressant actions also possess antioxidant properties, which reinforces the notion of the relationship between oxidative stress and depressive disorders (Mori-Okamoto et al., 2004; Naveen et al., 2013; Valdés-Sustaita et al., 2017, 2021; Moore et al., 2018; Estrada-Camarena et al., 2020; Ruiz-Sánchez et al., 2020).

Due to their antioxidant characteristics, some natural compounds are being used to treat depression (Pathak et al., 2013), an example of this are polyphenol compounds that modulate neurotransmitter systems (Pathak et al., 2013), and at the same time, induce anti-inflammatory, antiapoptotic (Han et al., 2006), antigenotoxic (Yaidikar et al., 2014), antimutagenic, and antioxidant effects (Han et al., 2006; Holzmann et al., 2015), as well as being able to protect against oxidative cell damage (Scalbert et al., 2005). Polyphenols are subdivided into various elements, such as ellagitannins, including punicalagin (PNCG) and ellagic acid (EA), among other compounds. Ellagitannins are found in various natural sources such as pomegranate (*Punica granatum*), widely consumed globally (Cerdá et al., 2003a; Girish et al., 2012). Recently, the antidepressant and anxiolytic-like actions of an aqueous extract of *P. granatum* enriched with ellagitanins (AEPG) were assessed in female rats (Estrada-Camarena et al., 2020). AEPG induced an antidepressant-like action after a subacute (Valdés-Sustaita et al., 2021) or chronic schedule of administration (Valdés-Sustaita et al., 2017). In addition, AEPG reduced the immobility behavior after an oral or intraperitoneal route of administration (Valdés-Sustaita et al., 2017, 2021), suggesting that their actions do not necessarily require the first step’s metabolism. In fact, its mechanism of action appears to be related to the activation of the estrogen receptor β, participation of the serotoninergic system, and the peroxisome proliferator-activated receptor (PPARγ) (Valdés-Sustaita et al., 2017, 2021; Estrada-Camarena et al., 2020); however, its activity as an antioxidant in ovariectomized rats is unknown. Aside from the main components found in the AEPG, only the antidepressant and antioxidant activity of EA has been demonstrated in male mice (Dhingra and Chhillar, 2012; Girish et al., 2012); however, there are no reports about the PNCG effect as an antidepressant or antioxidant in ovariectomized female rats.

Therefore, the present study aimed to evaluate whether AEPG, PNCG, and EA treatment prevent LPX, ROS formation, and cellular dysfunction in brain homogenates from ovariectomized rats exposed *ex vivo* to the pro-oxidants ferrous sulfate (FeSO_4_) and peroxynitrite (ONOO^–^). In addition, we evaluated whether PNCG and EA present in AEPG induce antidepressant-like effects. Additionally, their capacity to scavenge hydroxyl radical (^⋅^OH), superoxide anion (O_2_.), and ONOO^–^ was determined in chemical combinatorial assays.

## Materials and Methods

### Reagents

The AEPG was prepared from whole fruits kindly donated by Nutracitrus SL (Elche, Alicante, Spain). The dose was selected based on a previous study where the extract induced an antidepressant-like effect in ovariectomized rats (Valdés-Sustaita et al., 2017). PNCG and EA was obtained from Sigma Aldrich (St. Louis, MO, United States), malonaldehyde (MDA), 2’,7’-dichlorofluorescein (DCF), 2’,7’-dichlorodihydrofluorescein diacetate (DCF-DA), Ferrous Sulfate (FeSO_4_), ascorbic acid, nitroblue tetrazolium (NBT), phenazine methosulfate (PMS), ethylenediamine-tetra acetic acid (EDTA), nicotinamide adenine dinucleotide (NADH), *N*-2-piperazine hydroxy ’-2-ethane sulphonic acid (HEPES) and hydrogen peroxide (H_2_O_2_) were all obtained from Sigma Chemical Company (St. Louis, MO, United States). The solutions were prepared using deionized water from a MilliRQ purifier system (Millipore. Billerica, MA, United States).

### Animals

Ovariectomized female Wistar rats (250–300 g, 3 months old) were housed in groups of five per polycarbonate cage with food and water available *ad libitum*, maintained in a 12 h light/dark cycle (lights on at 21 h), in a temperature-controlled room at 22°C. Tissue samples were immediately collected after decapitation of the animals and were preserved at a temperature of −70°C. All experimental procedures with animals were carried out following the official Mexican standard for the care and handling of animals (NOM-062-ZOO-1999) and approved by the Ethics Committee of the National Institute of Psychiatry Ramón de la Fuente Muñiz, CEI-200 (December 10, 2015).

### Ovariectomy Surgical Procedures (OVX)

For the study, all the rats used were ovariectomized under anesthesia with tribromoethanol (2% dose 0.1 mL/kg, i.p.). For surgery, a single incision was made in the midline of the ventral region, and oviducts were exposed. The ovaries were removed, which was corroborated by visual inspection. After 3 weeks, the animals were randomly assigned to each experimental and behavioral group (Bekku and Yoshimura, 2005; Estrada-Camarena et al., 2011).

### Experimental Design

**FIGURE 1:**
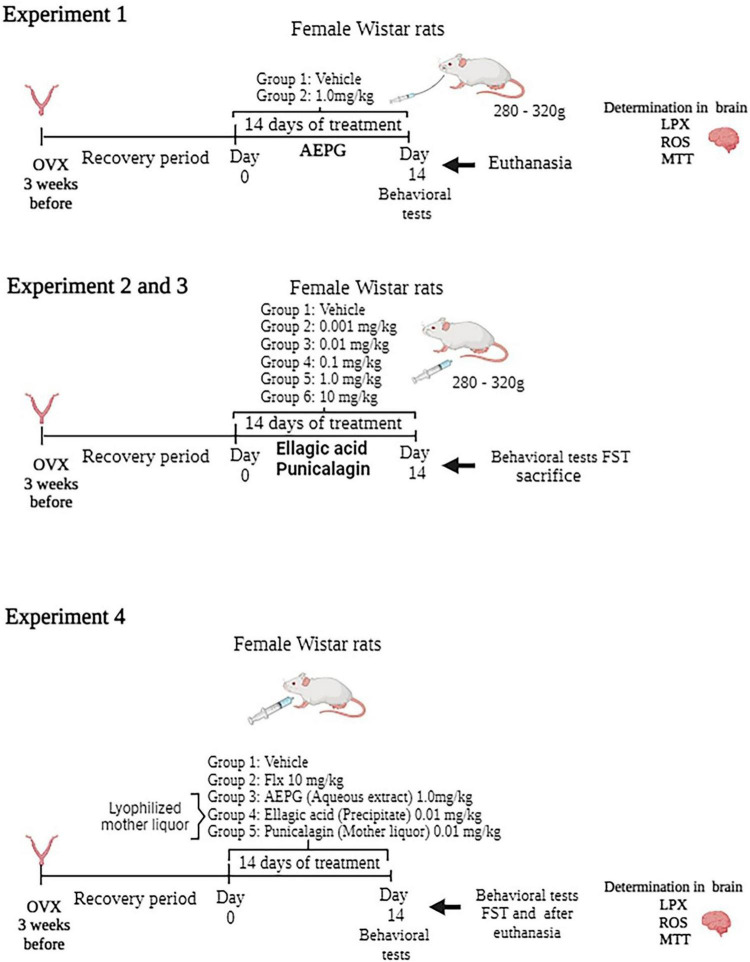
Timeline of the experimental series.

In experiment 1, determinations of LPX, ROS, as well as for the methyl thiazol tetrazolium (MTT) reduction assay in brains from rats treated with a daily intragastric administration of AEPG (1.0 mg/kg for 14 days in a volume of 2 mL/kg) were performed (Figure 1). The anti-immobility effect of the AEPG was previously published (Valdés-Sustaita et al., 2017). The AEPG was prepared from the whole fruit and dissolved in water. The dose selection was based on a previous study in which AEPG showed an antidepressant and anxiolytic-like effect in ovariectomized rats (Valdés-Sustaita et al., 2017; Estrada-Camarena et al., 2020). After behavioral tests, all rats were killed by decapitation, and the brains were immediately removed to be placed in vials for storage in a −80°C freezer for further study.

In experiments 2 and 3, a dose-response curve of commercial PNCG and EA (0.0010, 0.010, 0.10, 1.0 mg/kg) was constructed to evaluate their antidepressant-like effect in the forced swim test (FST) with general activity prior to FST using ovariectomized female Wistar rats. In a previous study, we observed similar results with AEPG after an intraperitoneal or intragastric administration (unpublished results) as well as after a subacute (three injections in 24 h) or 14 days of treatment (Valdés-Sustaita et al., 2017, 2021). For this reason, to evaluate whether EA and PNCG induce an anti-immobility effect in the FST, both compounds were intraperitoneally administered during 14 days/once a day (Figure 1). Each compound was dissolved in vehicle (saline solution 0.9% NaCl).

Finally, from AEPG, a lyophilized mother liquor that main contains PNCG and a precipitated solid that mainly contains EA. To corroborate the effect of PNCG and EA obtained from AEPG, in experiment 4, independent groups of ovariectomized rats were assigned to one of the following groups: vehicle (saline treatment), Fluoxetine (10 mg/kg), AEPG (1.0 mg/kg), EA (0.010 mg/kg) and PNCG (0.010 mg/kg). All treatments were administered i.p., for 14 days/once a day. Thirty minutes after of FST session, rats were euthanized, and brains were removed to be placed in vials for storage in a -80°C freezer for oxidative stress determination.

The presence of EA and PNCG in AEPG was determined by gas chromatography-mass spectrometry.

### General Activity Test

A general activity test session was performed on day 14 to discard if treatment-related alterations in locomotor activity may influence behaviors on FST test. The rats were gently placed in one of the corners of a rectangular acrylic cage (43 × 33 × 20 cm) with a grid drawn on the floor (12 squares of 11 × 11 cm). The number of times the animal crossed any square with its four legs (crossing) in 5 min was recorded. After each test session, the cage was thoroughly cleaned with a cleaning solution (Estrada-Camarena et al., 2003).

### Forced Swimming Test

The forced swim test (FST) was performed by exposing the rats to a glass cylinder (46 cm deep and 20 cm in diameter) with water at 23–25°C in which the rats could not touch the bottom or hold on with their legs or tail. Two swimming sessions were performed: an initial 15 min pretest session on treatment day 13, 1 h after drug administration. On day 14, the rats received their treatment, and 60 min later, they were again subjected to the forced swim test for 5 min (test session). After each swimming session, the rats were removed from the cylinders, dried with paper towels, placed in warm cages for 30 min, and returned to their cages. The test sessions were held between 12:00 and 15:00 which were video-recorded for later scoring. A single observer, blind to the treatment conditions, performed the score analysis counting the number of episodes of immobility, swimming, or climbing behavior shown by rats in intervals of 5 s during 5 min (Porsolt et al., 1977, 1978).

Three behaviors scores were defined as follow: (Scapagnini et al., 2012) immobility, where the rat floated in the water without fighting and did only the movements necessary to keep the head above the water; (Nestler et al., 2002) swimming, rat shows active swimming movements, more than necessary to simply keep the head above the water, such as moving around the cylinder or diving; and (Yan et al., 2010) climbing, rat shows active front leg movements in and out of the water, usually directed against the walls (Estrada-Camarena et al., 2003; Cruz et al., 2009).

### *Ex vivo* Assays

Brain tissue from rats treated with AEPG, EA, PNCG (14 days, i.p.), or vehicle was homogenized (1:10 w/v) in Krebs buffer (pH 7.4). Then, 375 μL of each brain homogenate was incubated alone or with FeSO_4_ (10 μM), or ONOO^–^ (50 μM) in a final volume of 500 μL for 2 h at 37°C in a water bath. After incubation, the sample was used to determine ROS, LP, and MTT reduction simultaneously.

### Lipid Peroxidation in Brain and Liver Homogenates

After incubation with the pro-oxidants FeSO_4_ (5 μM) and ONOO^–^ (25 μM), 500 μL of the TBA reagent (containing 0.75 g of TBA + 15 g of trichloroacetic acid + 2.54 mL of HCl) was added, and the final solutions were re-incubated in a boiling water bath (94°C) for 20 min. Samples were then kept on ice and centrifuged at 12,000 *g* for 5 min. MDA was determined as a colorimetric product using Synergy™ HTX multi-mode microplate reader (Biotek Instruments, Winooski, VT, United States) at a wavelength of 532 nm. MDA concentrations were calculated by interpolation on an MDA standard curve, constructed in parallel. Protein determinations were made by the Lowry method (Lowry et al., 1951). The results were expressed as μmoles of MDA per milligram of tissue.

### Determination of Reactive Oxygen Species

Reactive oxygen species (ROS) were determined through DCF-DA oxidation (Ali et al., 1992; Herrera-Mundo and Sitges, 2010). Briefly, 125 μL of forebrain and liver homogenates previously incubated with FeSO_4_ and ONOO^–^ were mixed with DCF-DA solution (final concentration: 75 μM); then, incubated in dark conditions at 37°C for 30 min. After incubation, the samples were centrifuged at 9,000 *g* for 10 min. ROS were detected in supernatants using fluorescence spectrophotometry (Synergy™ HTX multi-mode microplate reader, Biotek Instruments) at an excitation wavelength of 480 nm and an emission wavelength of 521 nm. Results are expressed as a percentage of ROS production considering the control as 100%.

### Assay of Methyl Thiazol Tetrazolium

The cellular function was assessed in forebrain and liver homogenates by determining MTT reduction to formazan crystals. Briefly, the brain homogenate (125 μL) was mixed with 4 μL of the MTT (5 mg/mL) and then incubated for 15 min at 37°C. Samples were then centrifuged at 17,000 *g* for 3 min and the pellets were suspended in 250 μL with acid-isopropanol. Optical density was determined using an Eon microplate reader (Biotek Instruments) at a wavelength of 570 nm. Results are expressed as the percentage of MTT reduction considering the control as 100%.

### Protein Assay

Protein was determined according to the Lowry method (PMID 14907713) using bovine serum albumin as a protein standard.

### ^⋅^OH Scavenging Activity Assay

The ability of the study compounds (AEPG 0–4 mg/mL, PNCG 0–4 mg/mL, and EA 0–20 mg/mL) to scavenge hydroxyl radical (^⋅^OH) was estimated through the Fe^3^ + -EDTA-H_2_O_2_-deoxyribose system (Halliwell et al., 1987; Floriano-Sánchez et al., 2006). The system contained different concentrations of AEPG, PNCG, and EA or an equivalent volume of vehicle (distilled water) for the control, 0.2 mM ascorbic acid, 0.2 mM FeCl_3_, 0.208 mM EDTA, 1 mM H_2_O_2_, 0.56 mM deoxyribose, and 20 mM phosphate buffer (pH 7.4). ^⋅^OH was generated by incubating the mixture at 37°C for 60 min. The iron salt (FeCl_3_) was mixed with EDTA prior to its addition to the reaction mixture. The degree of degradation of deoxyribose by the ^⋅^OH formed was measured directly in the aqueous phase by the thiobarbituric acid test (TBA). Briefly, 250 μL of the TBA solution was added to the samples and boiled in a water bath (94°C) for 10 min. Optical density was estimated at a wavelength of 532 nm using a Synergy™ HTX multi-mode microplate reader (Biotek Instruments). The results are shown as percent of OH scavenging capacity.

### O_2_^⋅⁣–^ Scavenging Assessment

O_2_^⋅⁣–^ scavenging was evaluated according to the method previously published (Fontana et al., 2001) based on the reduction of nitroblue tetrazolium (NBT) salt. The non-enzymatic PMS/NADH system that generates O2^⋅⁣–^ was used to reduce NBT into purple-colored formazan. The reaction mixture contained HEPES buffer (20 mM, pH 7.2), 196 μM NADH, 39.2 μM NBT, 3.92 μM PMS, and increasing concentrations of AEPG (0–1 mg/mL), EA (0–2 mg/mL), PNCG (0–1 mg/mL). The final volume of the mixture was 130 μL. The optical density was determined at a wavelength of 560 nm in a Synergy™ HTX multi-mode microplate reader (Biotek Instruments) each minute for 3 min. All tests were carried out in triplicate and independently. Results are expressed as a percentage of O2^⋅⁣–^ scavenging capacity.

### ONOO^–^ Scavenging Assay

ONOO^–^ was synthesized as previously described (Beckman et al., 1994). Briefly, 5 mL of an acidic solution (0.6 M HCl) of H_2_O_2_ (0.7 M) was mixed with 5 mL of 0.6 M KNO_2_ in an ice bath for 1 s, and the reaction was quenched with 5 mL of ice-cold 1.2 M NaOH. Residual H_2_O_2_ was removed using granular MnO_2_ pre-washed with 1.2 M NaOH, and the reaction mixture was left overnight at -20°C. The resulting yellow liquid layer on top of the frozen mixture was collected for the experiment. ONOO^–^ concentration was determined before each experiment at 302 nm using a molar extinction coefficient of 1,670 M^–1^ cm^–1^. The ONOO^–^ scavenging activity was measured by monitoring the oxidation of DCF-DA to DCF by the modified method of Crow and Beckman (1996). The reaction mixture (in a final volume of 1.15 μL) contained 14 μM DTPA, 36.2 μM DCFH-DA; samples were incubated with different concentrations of AEPG (0–5 mg/mL), EA (0–20 mg/mL), PNCG (0–1 mg/mL) and exposed to 35 μM ONOO^–^. The optical density at 500 nm was determined in a Synergy™ HTX multi-mode microplate reader (Biotek Instruments). A test containing the reaction mixture, but not the sample, was considered as the 0% trapping capacity, or 100% of the induced DCF-DA oxidation by ONOO^–^ when added to the assay. To calculate the scavenging capacity of ONOO^–^, the readings of all the tubes with the various study compounds were expressed as percentage of oxidation of DCF-DA and converted to the percentage of scavenging using the tube with 100% oxidation as a reference with DCF-DA.

### Ultra High-Performance Liquid Chromatography

Samples were dissolved in water or methanol (HPLC grade) and filtered through 0.22 μm filters (GHP, Acrodisc 13, Waters) before being injected into the chromatograph. The ultra high-performance liquid chromatography (UPLC) technique was carried out in a Waters Acquity UPLC H-Class liquid chromatography equipment fitted with a Waters photodiode array detector (UPLC, Acquity Waters, Singapore) and the Empower chromatographic software version 3 (Waters, Milford, MA, United States). Sample analysis was performed with a Symmetry C-18 column (100Å, 150 mm × 4.6 mm, 5 mm, Waters, Ireland) with the thermostat at 40°C. The mobile phase consisted in a gradient system of Milli-Q water acidified with 0.3% formic acid (solvent A) and methanol (solvent B). The initial gradient mixture was 90% A: 10% B up to 5 min. Then, a linear 70% A: 30% B was applied from 5 min. Whereas methanol (B) was gradually increased to 100% in the time of 10–12 min. Finally, the gradient was returned to the initial concentrations (90% A: 10% B). A constant flow rate of 0.9 mL/min was considered during a total time of 12 min. The detection wavelength used was 254 nm. Commercial standards of PNCG and EA were used as reference for comparison of the retention times and maximum absorption wavelength data.

### Statistics

Reactive oxygen species, LPX, and MTT data are expressed as mean values ± S.E.M. The Sigma plot 12.0 program (version 12.3, Systat software) was used for the statistical analysis, using U-Mann-Whitney for specific comparisons. For behavioral data, a one-way ANOVA test was applied, followed by the Holm-Sidak test as *post-hoc* analysis, accepting as significant only values of *p* ≤ 0.05. The trapping capacity was expressed as 50% of the inhibitory concentration (IC_50_), which denotes the concentration of the compounds (AEPG, PNCG, and EA, in mg/mL) necessary to achieve a reduction of 50% on the respective molecules in oxidation relative to the test without the compounds of interest. The lowest IC_50_ value suggests the best elimination capacity of the compound.

## Results

### Aqueous Extract of *Punica granatum* Decreases Reactive Oxygen Species Formation by FeSO_4_ and ONOO^–^

**FIGURE 2:**
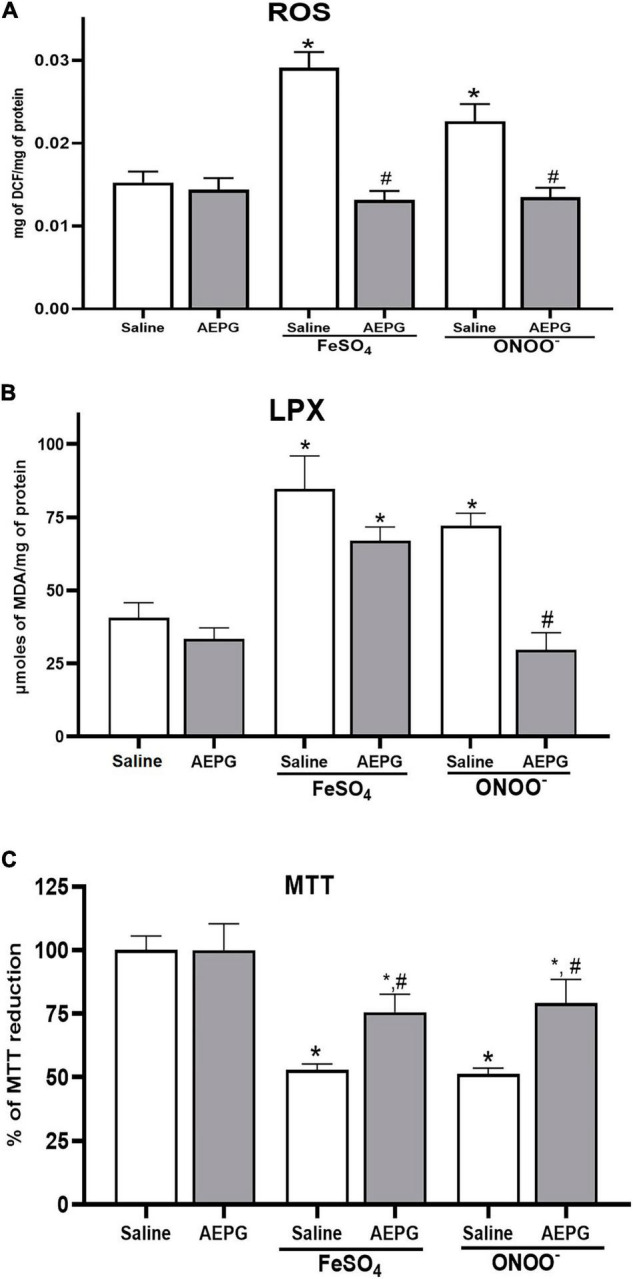
Effect of AEPG, on reactive oxygen species (ROS) **(A)**, Lipid peroxidation (LPX) **(B)** production and cellular dysfunction (MTT) **(C)** induced by FeSO_4_ (10 μM) and peroxynitrite (ONOO^–^; 50 μM) in brain homogenates from ovariectomized rats. Data are shown as mean ± S.E.M. of five experiments per group (each experiment corresponding to one rat). One-Way Anova test followed by *p < 0.05 vs control-group; ^#^versus vehicle (saline-group) for each pro-oxidant.

First, the AEPG treatment effect was evaluated when the brain homogenates were exposed *ex vivo* to pro-oxidants (FeSO_4_ and ONOO^–^). FeSO_4_ and ONOO^–^ induced a significant increase (91 ± 12.4 and 50 ± 23%, respectively) in ROS formation in brain homogenates compared to the control group (*p* < 0.05; 0.0153 ± 0.001 mg of DCF/mg protein) (Figure 2A). However, when rats were treated with AEPG (1.0 mg/kg, 14 days) and the brain homogenates were exposed *ex vivo* to the pro-oxidants, no effect was observed on ROS production (*p* < 0.05), indicating that AEPG prevented the oxidative damage induced by the pro-oxidants. One Way ANOVA values yielding the following values *F*_(3,14)_ = 7-0.57, *p* = 0.003.

### Aqueous Extract of *Punica granatum* Reduced the Lipoperoxidation (LPX) Produced by FeSO_4_

Lipid peroxidation was determined as a marker of oxidative Damage. After AEPG pretreatment, the Brain homogenates were exposed to pro-oxidants. As shown in Figure 2B, LPX was enhanced in homogenates of the brain (109 ± 11.3% and 78 ± 4.2%, respectively) by FeSO_4_ and ONOO^–^ compared to the control (*p* < 0.05; 40.56 ± 5.2 μmoles of MDA/mg protein). However, the administration of AEPG significantly decreased the LPX induced by ONOO^–^ (*p* < 0.001), and a decreasing trend is observed with FeSO_4_ in the brain. One Way ANOVA values yielding the following values *F*_(3,14)_ = 23.42, *p* < 0.0001.

### Aqueous Extract of *Punica granatum* Prevents Mitochondrial Damage Caused by FeSO_4_ and ONOO^–^ in the Brain

The reduction of MTT was evaluated as an index of cellular function in the rat brain (Figure 2C). For this, homogenate preparations were exposed to toxic concentrations of FeSO_4_ and ONOO^–^, and a value of 100% represents the total capacity of MTT reduction by the cell (control), whereas in the groups treated with FeSO4 and ONOO^–^, a decrease in cellular functionality of 52.84 and 51.25%, respectively, was observed in the brain (*p* < 0.001). AEPG pretreatment partially recovered cellular dysfunction induced by the *ex vivo* exposure to FeSO4 and ONOO^–^ in rats’ brains (22.7 and 28%, respectively; *p* < 0.05). One Way ANOVA values yielding the following values *F*_(2,10)_ = 11.06, *p* = 0.002.

### General Locomotor Activity

Table 1 shows the effect of FLX, AEPG, EA, and PNCG on general activity. As it can be seen, none of the treatments significantly modify the locomotor activity test [*F*_(4,33)_ = 0.35, ns], discarding that an alteration of a stimulatory action of any treatment could contribute to decreasing the immobility behavior in FST.

**TABLE 1:** Effect of aqueous extract of FLX, AEPG, fractions with PNCG and EA on general motor activity.

Square		

Treatment	Crossed/5 min	(*n*)
CTL	44.16 ± 5.22	8
FLX	55.28 ± 5.14	8
AEPG	49.17 ± 7.87	7
EA	48.50 ± 4.67	8
PNCG	53.37 ± 5.78	8

*Mean ± standard error. CTL = Control; FLX = Fluoxetine 10 mg/kg; AEPG = aqueos extract of Punica granatum (1.0 mg/kg); EA = ellagic acid (0.01 mg/kg); PNCG = punicalagin (0.01 mg/kg).*

### Punicalagin and Ellagic Acid Induce Antidepressant-Like Effects in Ovariectomized Rats

**FIGURE 3:**
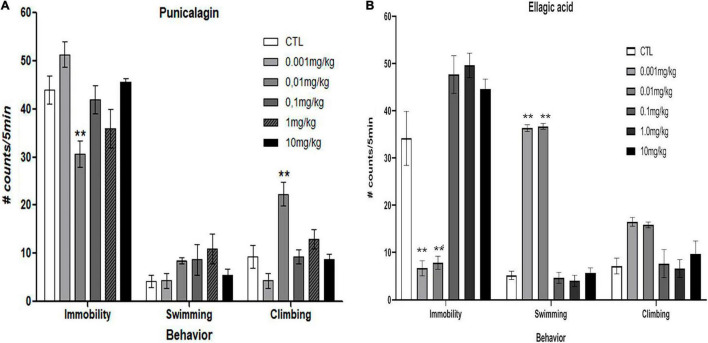
Effect of chronic administration (0.001, 0.01, 0.1, 1.0, and 10 mg/kg, i.p, once a day/14 days) of commercial punicalagin [panel **(A)**, *n* = 7 per dose] and commercial ellagic acid [panel **(B)**, *n* = 7] in ovariectomized rats subjected to forced swimming test. Data are presented as the mean (S.E.M.) of the number of counts of immobility, swimming, and climbing behavior in a 5-min test session. Comparisons vs. control group (saline solution). One-way ANOVA test **p < 0.001 versus control group.

Figure 3A shows that commercial PNCG decreased the immobility behavior compared with the control group at a dose of 0.01 mg/kg (*p* < 0.03) [*F*_(5,37)_ = 4.814, *p* = 0.002], In addition, PNCG produced a significant increase in the climbing behavior at 0.01 mg/kg (*p* < 0.001) (*F*_5,37_ = 8.625, *p* < 0.001) without modification of the swimming behavior [*F*_(5,37)_ = 1.242, *p* = 0.309].

Figure 3B shows that commercial EA at doses of 0.001 and 0.01 mg/kg produced a significant reduction in the immobility behavior (*p* < 0.001) compared to the control group [*F*_(5,57)_ = 15.733, *p* < 0.001]. Regarding the effect of EA on the swimming behavior, a significant increase of the swimming behavior can be observed (*p* < 0.001) [*F*_(5,57)_ = 17.144, *p* < 0.001], panel B (Figure 3) without promoting significant changes in the climbing behavior [*F*_(5,57)_ = 3.541, *p* = 0.007].

### Scavenging Activity of Aqueous Extract of *Punica granatum*, Punicalagin, and Ellagic Acid in Combinatorial Chemistry Assays

**FIGURE 4:**
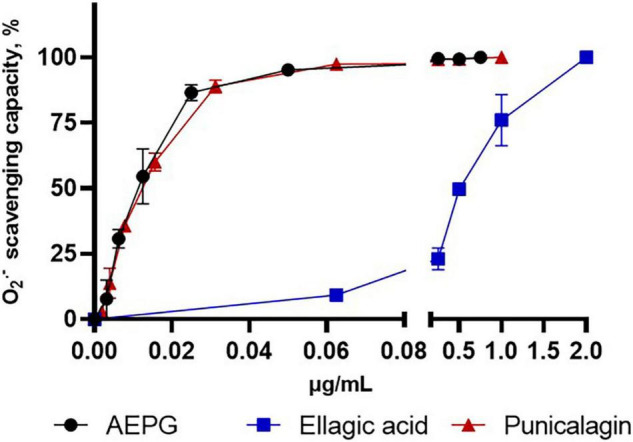
Superoxide scavenging capacity of AEP, commercial punicalagin, and ellagic acid in a chemical combinatorial system. Data are represented as mean values ± S.E.M. of four to six experiments per concentration.

The redox profiles of AEPG, PNCG, and EA were determined using non-tissue synthetic systems, in which ROS were specifically generated through combinatorial chemistry assays. Figure 4 shows the superoxide scavenging effect of these compounds, being the AEPG and PNCG more effective than EA, since the IC_50_ for ROS was 0.012 and 0.015 μg/mL for AEPG and PNCG, respectively, while for EA, the IC_50_ was 0.49 μg/mL (Table 2).

**TABLE 2:** Scavenging capacity of different compounds against hydroxyl radical (OH), superoxide anion (O_2_^–^), and peroxynitrite (ONOO^–^).

IC_50_ (μg/ml)			

Compounds	^⋅^OH	O_2_^⋅⁣–^	ONOO^–^
AEPG	0.361 ± 0.0073	0.012 ± 0.001	1.334 ± 0.21
Ellagic acid	NA ^(0–20 mg/ml)^	0.4983 ± 0.004	NA ^(0–20 mg/ml)^
Punicalagin	0.422 ± 0.0244	0.015 ± 0.002	0.247 ± 0.02

*Values are expressed as IC_50_ (μg/mL).*

*IC_50_ is the inhibitory concentration, in vitro, to decrease in 50% the amount of reactive species in the tested media (mean ± standard error of the mean). n = 6–10.*

*NA, no activity was found up to the highest tested concentration (superscript).*

**FIGURE 5:**
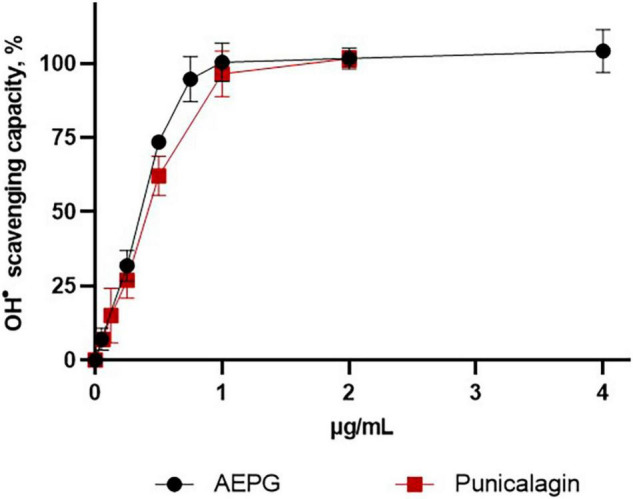
Hydroxide radical (OH)-scavenging capacity of AEPG, commercial punicalagin, and ellagic acid in a chemical combinatorial system. Ellagic acid had no effect. Data are represented as mean values ± S.E.M. of four to six experiments per concentration.

The hydroxyl radical (^⋅^OH)-scavenging capacity for AEPG, PNCG, and EA was tested (Figure 5), considering that this radical is one of the strongest oxidants that is also produced by FeSO_4_, in which AEPG demonstrated antioxidants effects previously. In this case, AEPG and PNCG were able to trap the ^⋅^OH radical with an IC_50_ 0.36 and 0.42 μg/mL, respectively. However, EA was not able to scavenge the hydroxyl radical at the concentration tested (0–20 mg/mL).

**FIGURE 6:**
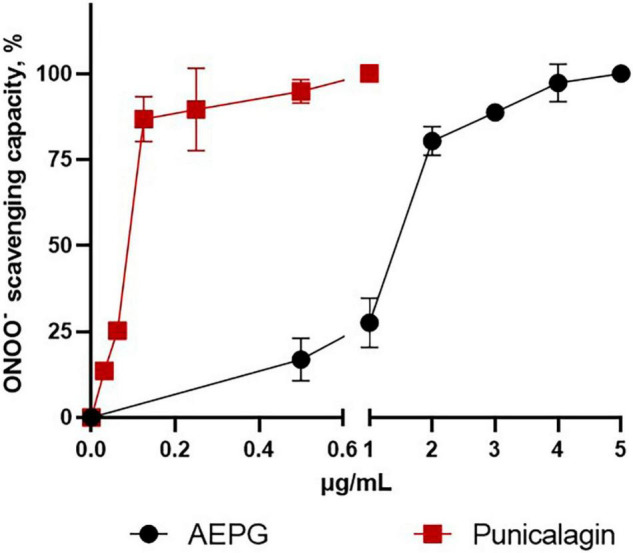
Peroxynitrite (ONOO^–^)-scavenging capacity of AEPG, commercial punicalagin, and ellagic acid in a chemical combinatorial system. Ellagic acid had no effect. Data are represented as mean values ± S.E.M. of three to five experiments per concentration.

Regarding peroxynitrite (ONOO^–^)-scavenging capacity (Figure 6), PNCG showed an IC_50_ of 0.2467 μg/mL, whereas AEPG was able to trap ONOO- but with a higher IC_50_ (1.33 μg/mL) than PNCG (Table 2). EA had no effect on trapping ONOO^–^ in the concentration range tested in this study (0–20 μg/mL).

### UPLC Chromatographic Profile of Aqueous Extract and Fractions From *Punica granatum*

**FIGURE 7:**
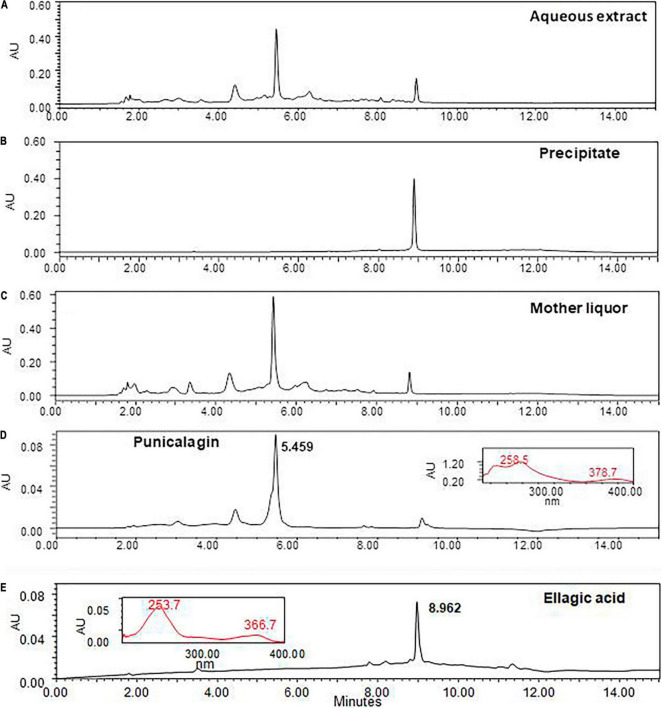
Chromatographic profile of the *Punica granatum* aqueous extract (AEPG) and fractions. The AEPG fruit [panel **(A)**, 10 mg/mL] and its fractions from precipitated solids [panel **(B)**, 2 mg/mL] and liophylized of mother liquor [panel **(C)**, 2 mg/mL] were analyzed by UPLC technique to determine the presence of punicalagin [panel **(D)**, retention time 5.459 min], and ellagic acid [panel **(E)**, retention time 8.962 min] as two of the most abundant constituents.

Chromatographic profile of the AEPG (10 mg/mL) (Figure 7A) allowed observing two major peaks at the retention time of 5.459 and 8.962 min corresponding to the PNCG and EA constituents, respectively, which were also supported by their maximum absorption wavelength data (inserted panels) of the corresponding standards of PNCG (258.5/378.7 nm, Figure 7D) and EA (253.7/366.7 nm, Figure 7E). These references were also considered to characterize the presence of both compounds in the lyophilized mother liquor (Figure 7C) and precipitated solids (Figure 7B) as PNCG and EA, respectively.

### Anti-Immobility and Antioxidant Brain Effect of Aqueous Extract of *Punica granatum* and Fractions

**FIGURE 8:**
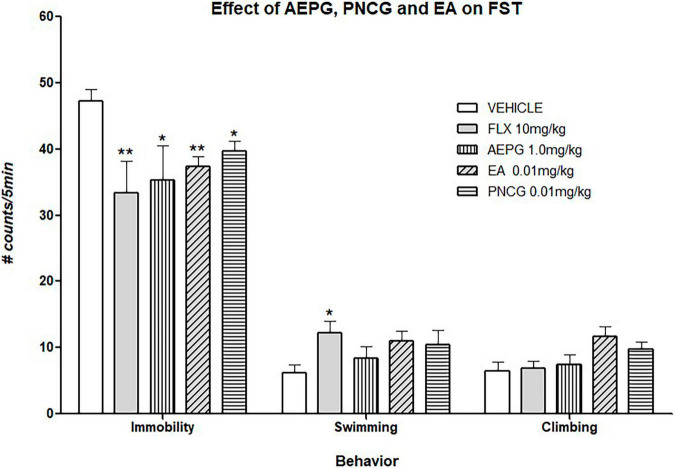
Effect of chronic treatment of AEPG and the fractions that contain EA and PNCG on ovariectomized rats subjected to forced swimming test. FLX antidepressant was used as positive control. Data are presented as the mean (S.E.M.) of the number of counts of immobility, swimming, and climbing behavior in a 5-min test session. Comparisons vs. control group (saline solution). One-way ANOVA test **p < 0.005, *p < 0.05 versus control group. AEPG = aqueous extract of *Punica granatum* (1.0 mg/kg), EA = ellagic acid (0.01 mg/kg), PNCG = punicalagin (0.01 mg/kg). FLX = fluoxetine (10 mg/kg).

The effect of AEPG and its lyophilized fractions were evaluated in the FST. As it can be noted in Figure 8, the treatment for 14 days with AEPG (1 mg/kg), the fraction that main contain PNCG (0.01 mg/kg) as well as that in which EA (0.01 mg/kg) was detected reduce the immobility behavior (*p* < 0.05) when compared with the control group. These effects were similar to that Induced by the antidepressant FLX [*F*_(4,33)_ = 7.41, *p* < 0.001]. The effect on swimming behavior is shown in Figure 8. As it can be seen, FLX increased significantly swimming behavior when compared against the control group (*p* < 0.05), while PNCG and EA showed a tendency to increase swimming behavior (*p* = 0.07). One Way ANOVA values yielding the following values [*F*_(4,33)_ = 3.32, *p* = 0.02]. Neither treatment significantly modify the climbing behavior [*F*_(4,33)_ = 2.51, *p* = 0.06].

### Aqueous Extract of *Punica granatum*, Ellagic Acid, and Punicalagin Decrease Reactive Oxygen Species and Lipid Peroxidation Formation Induced by FeSO_4_ and ONOO^–^

**FIGURE 9:**
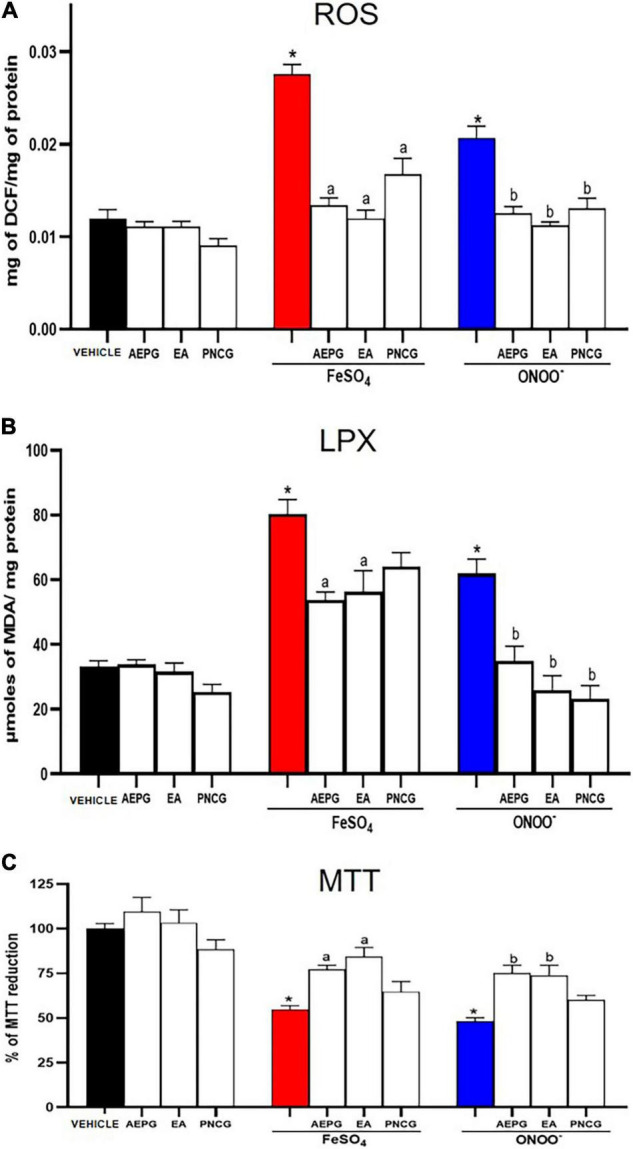
Effect of AEPG, fractions with EA and PNCG on reactive oxygen species (ROS, panel **A**), Lipid peroxidation (LPX, panel **B**) production and cellular dysfunction (MTT, panel **C**) induced by FeSO_4_ (10 μM), and peroxynitrite (ONOO^–^; 50 μM) in brain homogenates from ovariectomized rats. Data are shown as mean ± S.E.M. of seven experiments per group (each experiment corresponding to one rat). *p < 0.05 vs. control, ^a,b^*p* < 0.05 versus respective vehicle treated group based on the Mann-Whitney test for each pro-oxidant. AEPG = aqueous extract of *Punica granatum* (1.0 mg/kg), EA = ellagic acid (0.01 mg/kg), PNCG = punicalagin (0.01 mg/kg).

Figure 9A shows that the pro-oxidants ONOO^–^ and FeSO_4_ produced a significant increase in ROS formation in the brain of rats treated with vehicle (saline treatment) (*p* < 0.05). In contrast, in the rat brains treated with AEPG (1.0 mg/kg) and its fractions, EA (0.01 mg/kg) and PNCG (0.01 mg/kg), the pro-oxidant compounds ONOO^–^ and FeSO_4_ had no effect on ROS production (*p* < 0.05 versus respective control).

When determining LPX in brains of rats treated with AEPG, AE, PNCG, and subsequent exposure to pro-oxidant compounds (ONOO^–^ and FeSO_4_), we observed a significant decrease in LPX with AEPG (1.0 mg/kg) and EA (0.01 mg/kg) despite being subjected to FeSO_4_ or ONOO^–^ (*p* < 0.05 versus control group), Figure 9B.

### Aqueous Extract of *Punica granatum* and Ellagic Acid Prevents Cell Damage Induced by FeSO_4_ and ONOO^–^

When determining the percentage of reduction of MTT in brains treated with FeSO_4_ and ONOO^–^ we observed a cellular functionality decrease (54.64%, red column) and (48.08%, blue column) when compared with the control group (*p* < 0.05), Figure 9C. In contrast, this decrease was not observed in the brains of rats treated with AEPG and EA (*p* < 0.05). As it can be seen, the cellular functionality of the brains of rats treated with PNCG did not reach statistical significance.

## Discussion

Currently, pomegranate fruit is consumed all over the world, and various health benefits are attributed to it. The fruit contains mainly anthocyanins and ellagitannins, but is also rich in gallotannins, colorless flavonoids, and lignans, among other (poly) phenolic compounds (Fischer et al., 2011; Mena et al., 2012). It has been considered as one of the plants with the highest antioxidant values (Abdel Moneim, 2012). Actually, most studies aimed at studying the benefits of pomegranate have focused on juices or polyphenol extracts like the AEPG, which contain two main compounds: PNCG and EA (González-Trujano et al., 2015 and present study) that have been shown to exhibit high antioxidant capacities (Gil et al., 2000; Spilmont et al., 2014). According to this evidence, in our study, we demonstrated the scavenging capacity of AEPG, PNCG, and EA in chemical combinatorial assays, in which PNCG was the most effective scavenger compound as it was able to trap O_2_^⋅⁣–^, ^⋅^OH, and ONOO^–^. AEPG and PNCG showed greater efficiency in eliminating the O_2_^–^ and.OH radicals. It is worth mentioning that PNCG showed the lowest IC_50_ for ONOO^–^, which makes this compound an attractive scavenger of these radicals with almost 10-times more effectiveness than an endogenous antioxidant, such as GSH, under similar experimental conditions as in this work (Lugo-Huitrón et al., 2011). Regarding EA, it was not possible to determine its scavenger activity; thus, further experiments are needed to determine its effect.

Additionally, AEPG treatment showed great capacity to decrease the ROS and LPX in ovariectomized rats’ brains induced by the exposure of FeSO_4_ and ONOO^–^ *ex vivo.* It is known that ROS contribute to the development of cancer, neurodegenerative diseases, and the aging process, among others; it has also been established that LPX is one of the endpoints resulting from the formation of ROS in cells and tissues characterized by an imbalance in the production of these reactive species and antioxidant defenses, resulting in cellular and molecular damage (Basaga, 1990; Fedorova et al., 2013). These results are in line with those obtained in C57BL/6J × C57BBAJ mice that develop significant atherosclerosis by reducing LPX and oxidative stress (Rosenblat et al., 2006). The capacity to trap free radicals observed *in vitro* could be one of the mechanisms by which AEPG exerts its beneficial effects. The activity of AEPG and its compound PNCG as radical scavengers can contribute to preventing or improving these disorders related to oxidative stress, preserving homeostasis.

Regarding oxidative stress, the changes in the oxidative balance are directly involved in the pathogenesis of depressive disorders (Maes et al., 2011). The brain is especially vulnerable to oxidative stress due to its high metabolic rate and high consumption of oxygen (Valko et al., 2007; Maes et al., 2011; Di Lorenzo et al., 2016), in addition to the effects of LPX because polyunsaturated fatty acids are the main component of neuronal cell membranes, which are highly pro-oxidants. Furthermore, we showed in a rat model with OVX an increase in the percentage of MTT reduction by treatment with AEPG and EA. Thus, we can suggest that this extract could have a protective effect on the reducing capacity of mitochondria in the brains of rats exposed to toxic substances such as FeSO_4_ and ONOO^–^. It is important to note the fact that the PNCG acts as a potent scavenger while EA protects against ROS and LPX. Therefore, both compounds apparently by different mechanisms could contribute to exert the neuroprotective effects on AEPG. Nevertheless, it is of great importance to carry out detailed studies to allow us to rule out or reinforce this possible function.

On the other hand, it has been demonstrated that AEPG, in addition to its redox activity, produces antidepressant and anxiolytic-like actions (Valdés-Sustaita et al., 2017; Estrada-Camarena et al., 2020). In the present study, the antidepressant activity of the main compounds of the AEPG extract was determined. EA and PNCG induced an antidepressant-like action in the FST at low doses (0.001 and 0.01 mg/kg), suggesting that these compounds are relevant for the biological activity of AEPG. In line with this idea, we observed that two fractions obtained from AEPG, one with PNCG and the other with EA, reduced immobility after 14 days of i.p. administration in a similar manner to the antidepressant FLX. These results reinforce the notion that these compounds could be participating in the antidepressant-like action of AEPG. It is important to consider that other compounds no detected in the present study could act in synergy with PNCG and EA to induce the behavioral effects observed with AEPG and specific experiments are necessary to confirm this assumption.

Ellagic acid produced an antidepressant-like effect in ovariectomized rats subjected to the FST by reducing immobility behavior with a concomitant increase of swimming. These results agree with previous reports in mice (Dhingra and Chhillar, 2012; Lorigooini et al., 2019), albeit differences in doses were detected. In this sense, some of the discrepancies could be explained by the species used (rats versus mice), the via of administration, i.p versus p.o, and the sex of animals (female versus male). The behavioral profile produced by the ellagic acid suggests the participation of the serotonergic system (Girish et al., 2012; Valdés-Sustaita et al., 2021). Previous reports suggested the participation of the serotonergic system and estrogen receptor β as a mediator of the antidepressant-like action of AEPG (Valdés-Sustaita et al., 2017, 2021), and being EA one of the main components of this extract, it is possible that serotonin could be involved in its antidepressant-like action. According to this, Dhingra and Chhillar (2012) showed that serotonin depletion with *p*-chloro-phenyl-alanine partially cancels the antidepressant-like action of EA in mice. In addition, recently, the impact of this compound and urolitin A on tryptophan metabolism in humans and mice was measured (Yang et al., 2020). In mice, the treatment with EA but not urolitin A enhances indole propionate, decreases kynurenines (Yang et al., 2020), and increases serotonin brain levels, reinforcing the idea that ellagic acid acts on the serotonergic system to exert part of its effect as antidepressant.

Another non-excluding mechanism that could be participating in the antidepressant-like action of EA is related to its antioxidant properties. Dhingra and Chhillar (2012), showed that when mice are stressed, the main mechanism involved was the oxidative stress process by reducing the plasmatic nitrite levels (Dhingra and Chhillar, 2012) instead of the serotonergic mechanism. In addition, other authors showed the involvement of nitric oxide and NMDA receptors in the antidepressant-like action of EA in mice (Lorigooini et al., 2019). In the present study, ovariectomized rats were used, and OVX *per se* is a stressor since ovarian hormone deficiency increases ROS generation that may increase the oxidative stress markers (Fedorova et al., 2013; Yazğan et al., 2016; Zhou et al., 2021); hence, EA may confer specific protective antioxidant effects that also produce antidepressant-like actions. Albeit a limitation of the present study is that we did not include a sham group that permits us to discard if treatment is repairing the damage induced by OVX, the AEPG and the fraction that contain EA showed a clear protective effect against LPX and ROS formation in the rat brain exposed to pro-oxidants *ex vivo*.

Punicalagin produces an antidepressant-like effect decreasing immobility and enhancing the climbing behavior. As far as we know, this is the first report that analyzes the antidepressant-like action of this bioactive compound. Its mechanism of action is unknown, but several reports indicate the neuroprotective effect of PNCG *in vivo* and *in vitro* (Yaidikar et al., 2014). In line with this idea, in the present study, we detected a potent scavenger activity of PNCG and AEPG, and that fraction of the extract containing PNCG produced LPX reduction. In fact, administration of PNCG reverts the neuroinflammatory effect of LPS intervention in hippocampal slides by reducing pro-inflammatory cytokines TNFα and IL-6 (Olajide et al., 2014). Other authors also report its effect as a potent antioxidant in cell cultures (Clementi et al., 2018).

On the other hand, at higher doses (0.1, 1, and 10 mg/kg), ellagic acid and punicalagin did not modify the behavior in the FST, a result that could be related to a balance between the beneficial and harmful effects that occur with some natural compounds (Bouayed and Bohn, 2010), like exogenous antioxidants (Basaga, 1990; Bouayed and Bohn, 2010). From this point of view, it could be expected that some antioxidant compounds can also act as pro-oxidants, inhibiting the cellular antioxidant defense mechanism (Mazumder et al., 2019) and promoting toxic effects. However, no alterations in the behavior were detected at high doses, which is in line with other studies; for example, a study carried out in Sprague-Dawley rats in which the oral administration of PNCG for 37 days was not toxic (Cerdá et al., 2003b). The effects of these compounds may be unspecific for the FST, or the locomotor test and specific experiments are necessary to evaluate whether other systems can be affected.

The anti-immobility effect of PNCG and EA induced by the standards was accompanied by an increase in swimming or climbing behavior, which was not observed after the fractions of AEPG administration. The fact that in both cases, the anti-immobility effect is reproduced reinforces the notion of the active principles of the AEPG that contributes to its antidepressant-like action. Besides, the fact that the fractions administration did not modify the active behaviors in FST could be explained by the presence of other active compounds that veil their effects.

From the present data, it is not possible to conclude that the antidepressant-like effect observed after the treatment with AEPG and its active compounds are due to their antioxidant properties. However, the antioxidant effects observed in the brain animals tested in the FST permit us to consider as a plausible mechanism that could also contribute to behavior regulation. Specific experiments could contribute to elucidating if the mechanism of action involved in the antidepressant-like action of both active compounds of AEPG, PNCG, and EA, are associated with their neuroprotective effect.

A limitation of the present study is that we did not observe the trapping activity of the EA at the concentrations tested since the coloration emitted a wavelength that did not allow obtaining any reading in the equipment used for this determination. It would be of great help for future studies to develop a methodology allowing us to attenuate this coloration and make that determination.

In conclusion, AEPG, PNCG, and EA have an antioxidant capacity and antidepressant-like actions. Their ability as antioxidants could contribute to decreasing the prooxidant state induced by the stress and loss of ovarian function.

## Data Availability Statement

The raw data supporting the conclusions of this article will be made available by the authors, without undue reservation.

## Ethics Statement

The animal study was reviewed and approved by Ethics Committee of the National Institute of Psychiatry Ramón de la Fuente Muñiz.

## Author Contributions

EE-C designed the research, had primary responsibility for final content, and supervised the project. NC-A, GA-M, DR, MG-T, and VP conducted experimental series. EE-C, NC-A, MG-T, and VP analyzed the data, performed statistical analysis, prepared graphical data representation, and discussed the data. NC-A wrote the original draft. EE-C, VP, MG-T, and CL-R wrote–review and editing. All authors contributed to the article and approved the submitted version.

## Conflict of Interest

The authors declare that the research was conducted in the absence of any commercial or financial relationships that could be construed as a potential conflict of interest.

## Publisher’s Note

All claims expressed in this article are solely those of the authors and do not necessarily represent those of their affiliated organizations, or those of the publisher, the editors and the reviewers. Any product that may be evaluated in this article, or claim that may be made by its manufacturer, is not guaranteed or endorsed by the publisher.
